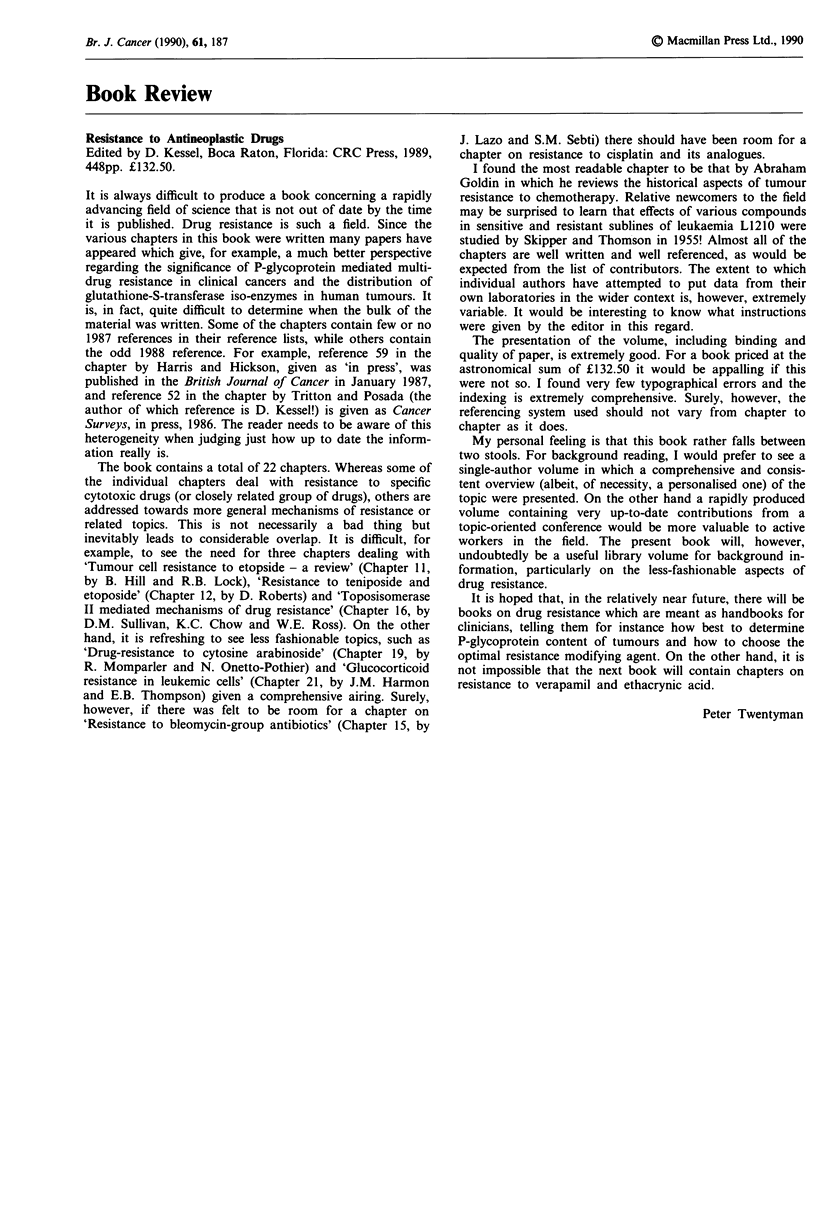# Resistance to antineoplastic drugs

**Published:** 1990-01

**Authors:** Peter Twentyman


*Br. J. Cancer* (1990), **61**, 187 © Macmillan Press Ltd., 1990

### Book Review

**Resistance to Antineoplastic Drugs**
Edited by D. Kessel, Boca Raton, Florida: CRC Press, 1989, 448pp. £132.50.

It is always difficult to produce a book concerning a rapidly advancing field of science that is not out of date by the time it is published. Drug resistance is such a field. Since the various chapters in this book were written many papers have appeared which give, for example, a much better perspective regarding the significance of P-glycoprotein mediated multidrug resistance in clinical cancers and the distribution of glutathione-S-transferase iso-enzymes in human tumours. It is, in fact, quite difficult to determine when the bulk of the material was written. Some of the chapters contain few or no 1987 references in their reference lists, while others contain the odd 1988 reference. For example, reference 59 in the chapter by Harris and Hickson, given as 'in press', was published in the *British Journal of Cancer* in January 1987, and reference 52 in the chapter by Tritton and Posada (the author of which reference is D. Kessel!) is given as *Cancer Surveys*, in press, 1986. The reader needs to be aware of this heterogeneity when judging just how up to date the information really is.

The book contains a total of 22 chapters. Whereas some of the individual chapters deal with resistance to specific cytotoxic drugs (or closely related group of drugs), others are addressed towards more general mechanisms of resistance or related topics. This is not necessarily a bad thing but inevitably leads to considerable overlap. It is difficult, for example, to see the need for three chapters dealing with 'Tumour cell resistance to etopside – a review' (Chapter 11, by B. Hill and R.B. Lock), 'Resistance to teniposide and etoposide' (Chapter 12, by D. Roberts) and 'Toposisomerase II mediated mechanisms of drug resistance' (Chapter 16, by D.M. Sullivan, K.C. Chow and W.E. Ross). On the other hand, it is refreshing to see less fashionable topics, such as 'Drug-resistance to cytosine arabinoside' (Chapter 19, by R. Momparler and N. Onetto-Pothier) and 'Glucocorticoid resistance in leukemic cells' (Chapter 21, by J.M. Harmon and E.B. Thompson) given a comprehensive airing. Surely, however, if there was felt to be room for a chapter on 'Resistance to bleomycin-group antibiotics' (Chapter 15, by J. Lazo and S.M. Sebti) there should have been room for a chapter on resistance to cisplatin and its analogues.

I found the most readable chapter to be that by Abraham Goldin in which he reviews the historical aspects of tumour resistance to chemotherapy. Relative newcomers to the field may be surprised to learn that effects of various compounds in sensitive and resistant sublines of leukaemia L1210 were studied by Skipper and Thomson in 1955! Almost all of the chapters are well written and well referenced, as would be expected from the list of contributors. The extent to which individual authors have attempted to put data from their own laboratories in the wider context is, however, extremely variable. It would be interesting to know what instructions were given by the editor in this regard.

The presentation of the volume, including binding and quality of paper, is extremely good. For a book priced at the astronomical sum of £132.50 it would be appalling if this were not so. I found very few typographical errors and the indexing is extremely comprehensive. Surely, however, the referencing system used should not vary from chapter to chapter as it does.

My personal feeling is that this book rather falls between two stools. For background reading, I would prefer to see a single-author volume in which a comprehensive and consistent overview (albeit, of necessity, a personalised one) of the topic were presented. On the other hand a rapidly produced volume containing very up-to-date contributions from a topic-oriented conference would be more valuable to active workers in the field. The present book will, however, undoubtedly be a useful library volume for background information, particularly on the less-fashionable aspects of drug resistance.

It is hoped that, in the relatively near future, there will be books on drug resistance which are meant as handbooks for clinicians, telling them for instance how best to determine P-glycoprotein content of tumours and how to choose the optimal resistance modifying agent. On the other hand, it is not impossible that the next book will contain chapters on resistance to verapamil and ethacrynic acid.

Peter Twentyman